# Clinical study on improving the function of female bladder in controlling urine by acupuncture Zhibian (BL54) under ultrasound guidance

**DOI:** 10.1007/s00345-024-05004-2

**Published:** 2024-05-06

**Authors:** Jinling Dai, Xiaojin Zhang, Feng Lian, Hong Li, Jie Tu, Yuelai Chen, Zhu Jin

**Affiliations:** 1 Shanghai University of Traditional Chinese Medicine, Shanghai, 201203 China; 2 Seventh People’s Hospital of Shanghai University of Traditional Chinese Medicine, Shanghai, 200137 China; 3 Longhua Hospital Shanghai University of Traditional Chinese Medicine, Shanghai, 570105 China

**Keywords:** Ultrasound, Acupuncture, BL54, Pudendal artery, Bladder

## Abstract

**Objective:**

To observe the effect of acupuncture Zhibian (BL54) on the function of the bladder in controlling urine in women under ultrasound.

**Method:**

74 healthy subjects were randomly divided into deep acupuncture group of 37 cases and shallow acupuncture group of 37 cases. Under the guidance of ultrasound, the two groups of subjects were acupunctured at bilateral BL54. The deep acupuncture group was acupunctured to the pudendal nerve, and the shallow acupuncture group was acupunctured to the superficial fascia. Ultrasound was used to observe the peak systolic velocity (PSV), time average maximum velocity (TAMX), end diastolic velocity (EDV), pulsation index (PI), resistance index (RI) of the pudendal arteries, and bladder volume of two groups of subjects before and after acupuncture. The anatomical hierarchical structure of bilateral BL54 and score of Chinese version of the Massachusetts General Hospital Acupuncture Sensation Scale (C-MASS) of all subjects was measured.

**Result:**

After acupuncture, the PSV, TMAX of the pudendal artery, bladder volume, and the Score of C-MASS Scale in the deep acupuncture group were higher than in the shallow acupuncture group (*P* < 0.05). The RI of the pudendal arteries in the shallow acupuncture group decreased compared to before acupuncture (*P* < 0.05).

**Conclusion:**

Acupuncture at the BL54 can increase the blood flow velocity of the pudendal artery, improve the function of the bladder in controlling urine in women, and different depths of acupuncture will have different therapeutic effects.

## Introduction

Acupuncture is an important branch of traditional Chinese medicine. It has the advantages of safety and convenience, and a good therapeutic effect on stress urinary incontinence (SUI) [1]. But the acupuncture method in clinical practice still lacks a unified standard, which It is also a problem that has always existed in the entire acupuncture treatment system, and also brings challenges to clinical practice and research [2, 3].

At present, ultrasound-guided acupuncture has been used clinically [4]. On the one hand, ultrasound can clearly display the anatomical structure the acupuncture points and improve the accuracy of acupuncture [5]; on the other hand, ultrasound can also provide reliable objective evaluation indicators for the efficacy of acupuncture [6]. Therefore, we dynamically observed the entire process of acupuncture under ultrasound, objectively analyzed the impact of acupuncture at BL54 on female bladder continence function. The report is as follows.

## Methods

### Study design

This was a randomized, controlled trial. Eligible subjects were recruited and treated at Seventh People's Hospital of Shanghai University of Traditional Chinese Medicine from 20 May 2023 to 31 December 2023. The study was performed according to the Declaration of Helsinki and approved by the Ethics Committee of the Seventh People's Hospital of Shanghai University of Traditional Chinese Medicine (batch number: 2023-7th-HIRB-022), and was registered at the China Clinical Trial Registration Center (registration number: ChiCTR2300071542).

### Inclusion criteria

(1) Past health, no history of urinary leakage, residual urine less than 15 ml [7]; (2) Aged 20–40 years old; (3) Have a history of childbirth, with ≤ 2 births, and normal cognitive ability; (4) Body mass index (BMI) 18.5—26.0 kg/m ^2^; (5) Voluntarily participate in this study and sign the informed consent form.

### Exclusion criteria

(1) People with skin infection at acupuncture points; (2) People with severe fear of needles, unable to cooperate or unwilling to accept acupuncture; (3)Those who have recently taken drugs that affect urinary function; (4) Those who have participated or are participating in other clinical studies within 3 months.

### Elimination criteria

All situations that violate the inclusion and exclusion criteria of this study.

### Sample size calculation

According to the preliminary test results of the research group, the mean (SD) PSV of the pudendal artery after acupuncture in the deep acupuncture group was 57.63 (10.36), and in the shallow acupuncture group was 52.35 (4.24). The G Power3.1.9.7 software was used for estimation. Two-sided test. It is calculated that at least 74 subject samples are required. Because this study only treated subjects once, there is no dropout or loss to follow-up. In the end, a total of 74 subjects were treated in this study.

### Randomization and blinding

Use the SPSS 25.0 random number generator and the visual binning function to divide all subjects randomly into 2 groups. There were 37 cases in each group. Data collectors and statistical analysts were not aware of the specific grouping, but it was open to acupuncturists.

### Interventions

The subjects were instructed to empty their bladders 40 min before acupuncture, and then not drink water or urinate until the end of acupuncture. Take bilateral BL54. The BL54 is located at the intersection of the upper 2/5 and lower 3/5 of the line connecting the medial edge of the posterior superior iliac spine and the medial edge of the greater trochanter of the femur.

Deep acupuncture group: The subjects were placed in the prone position, and the skin of the bilateral BL54 was wiped and disinfected with 75% ethanol, guided by color Doppler ultrasound (Netherlands PHILIPS company, Affiniti 70W model, frequency set to 3.0–6.0MHZ) Then, place the abdominal probe (frequency 1–5 MHz) on the skin above the BL54. Use a 0.35 mm × 100 mm stainless steel acupuncture needle at an angle of 45° with the skin and insert it obliquely toward the inside of the pudendal artery (the pudendal nerve and pudendal artery run parallel) for about 75—85 mm, and the needle tip reaches near the pudendal nerve. It is appropriate for the subject to feel numbness radiating to the perineum. The needle should be retained for 10 min.

Shallow acupuncture group: Use a 0.35 mm × 40 mm stainless steel acupuncture needle at an angle of 45° to the skin and insert the needle obliquely toward the inside of the pudendal artery for about 10–20 mm, and the needle tip reaches the superficial fascia above the pudendal nerve. The needle should be retained for 10 min. Both groups received acupuncture on the day of inclusion, with only one acupuncture.

### Outcome measures

#### Clinical primary outcome

Pudendal artery blood flow velocity: peak systolic velocity (PSV), time average maximum velocity (TAMX), end diastolic velocity (EDV), pulsatility index (PI), resistance index (RI).

The subject was placed in a prone position, breathing steadily, fully exposing the skin at the BL54 on both sides. The ultrasound abdominal probe was placed at the BL54 before and after acupuncture. The pudendal artery was found on the inside of the ischial spine, and the PSV, TAMX, EDV, PI and RI of the bilateral pudendal arteries of the two groups of subjects were measured.

#### Clinical secondary outcomes

(1) Bladder volume: The subject is placed in a supine position, and the skin at the projection of the bladder surface is fully exposed. The probe is placed on the lower abdomen, and subjects' bladder volumes were measured before and after acupuncture.

(2) Anatomical structure of the BL54 under ultrasound: The subject was placed in a prone position, fully exposing the skin of the BL54 on both sides, placing the ultrasonic high-frequency probe (frequency 5–12 MHz) on the skin of the BL54, and measuring The vertical distance from sebum, superficial fascia, deep fascia to the skin, and then use the abdominal probe to measure the vertical distance from the pudendal nerve to the skin (because the penetration of the high-frequency probe is weaker than the abdominal probe, the depth of the pudendal nerve cannot be observed).

(3) The Chinese version of the Massachusetts General Hospital Acupuncture Sensation Scale (C-MASS) [8, 9]: includes 12 questions, such as soreness, fullness, numbness, coldness and warmth, etc. The total score is 0–120. The higher the score, the stronger the acupuncture sensation.

### Statistical analysis

SPSS 25.0 statistical software was used for statistical analysis of data. Continuous variables were presented as mean values and standard deviations (SD). For measurement data that obeys the normal distribution, the two independent samples *t* test is used for comparison between groups*,* and for comparison within the group, the paired sample *t* test is used. For measurement data that does not obey the normal distribution, the Mann–Whitney U is used between groups, and Wilcoxon test within groups were used for analysis. All are two-sided tests. *P* < 0.05 is considered a statistically significant difference.

## Results

### Participants' characteristics

**Fig. 1:**
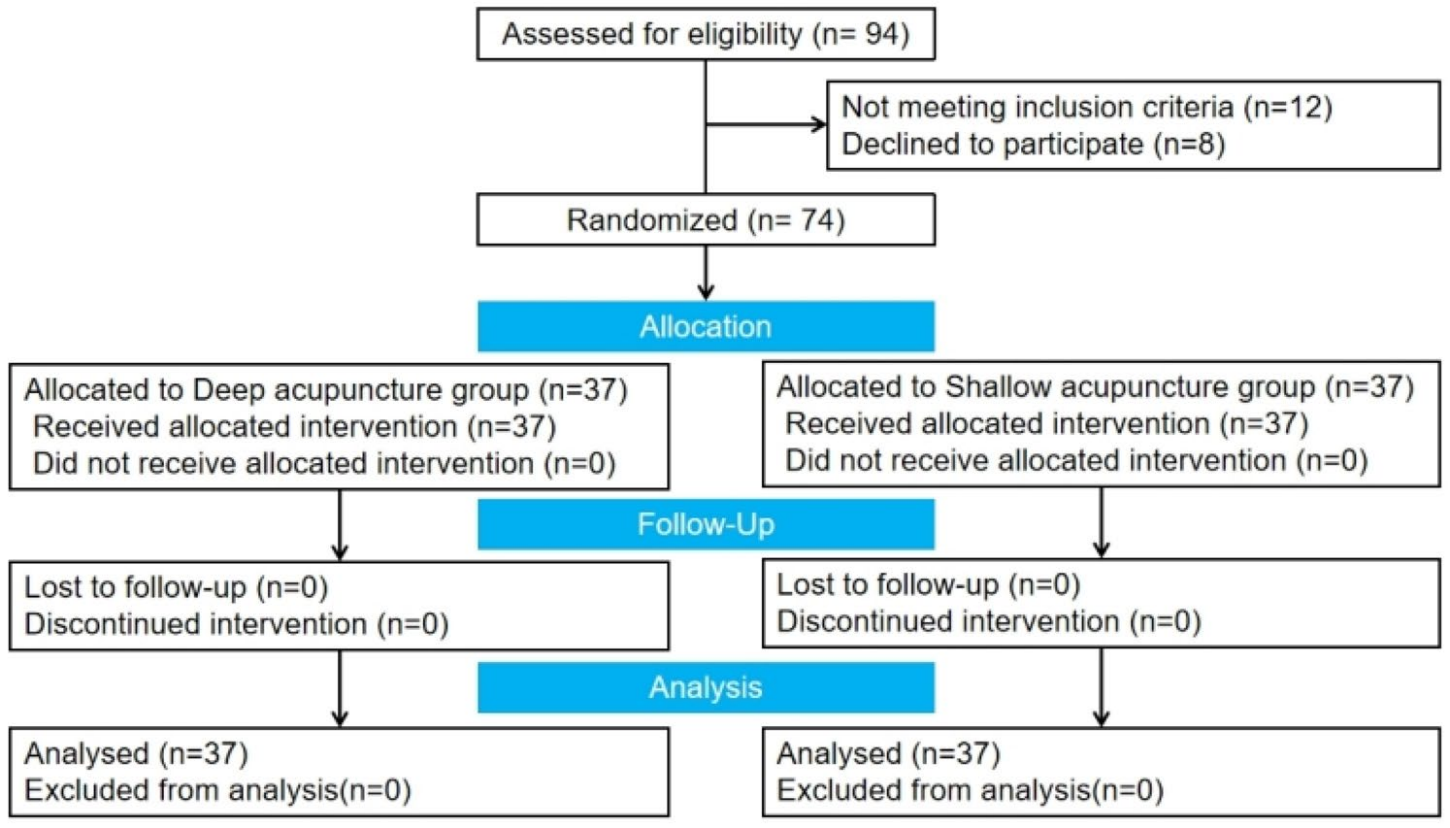
CONSORT flow diagram

The consort flow diagram of this study is shown in Fig. 1. Compare the age, height, weight, and BMI of the two groups of subjects, there was no statistically significant difference (*P* > 0.05) and they were comparable (Table 1).

**Table 1:** Comparison of general clinical data between two groups

Variables	Deep acupuncture group (*n* = 37)	Shallow acupuncture group (*n* = 37)	*P* value
Age, years	30.9 (4.63)	30.4 (5.31)	0.685
Height, cm	162.1 (4.12)	163.2 (4.13)	0.241
Weight, kg	61.4 (4.36)	60.7 (6.11)	0.567
BMI, kg/m^2^	23.4 (1.40)	22.8 (2.18)	0.178

Data are presented as mean (SD)

### Pudendal artery blood flow velocity

Before acupuncture, there was no statistically significant difference in the pudendal artery PSV, TMAX, EDV, PI, and RI of the two groups of subjects (*P* > 0.05), which was comparable. After acupuncture, the PSV and TMAX of the pudendal artery in the deep acupuncture group were significantly higher than those before acupuncture (*P* < 0.05), and the PSV and TMAX of the pudendal artery in the deep acupuncture group were greater than those in the shallow acupuncture group, and the difference was statistically significant (*P* < 0.05); In the shallow acupuncture group, the PSV of the pudendal artery was significantly higher than before acupuncture, and the RI of the pudendal artery was significantly lower than before acupuncture, and the difference was statistically significant (*P* < 0.05) (Table 2).

**Table 2:** Analytical statistics of pudendal artery blood flow and bladder volume before/after intervention between two groups

Variables		Deep acupuncture group (*n* = 37)	Shallow acupuncture group (*n* = 37)	*P* value
PSV, cm/s	Before intervention	39.96 (8.00)	41.50 (8.73)	0.429
	After intervention	52.55 (10.73)	47.55 (7.05)	0.018
TMAX, cm/s	Before intervention	8.63 (3.95)	10.13 (3.83)	0.101
	After intervention	12.54 (3.44)	10.95 (2.05)	0.018
EDV, cm/s	Before intervention	2.26 (3.20)	1.56 (2.24)	0.277
	After intervention	2.34 (3.21)	1.63 (2.51)	0.544
PI	Before intervention	5.62 (2.74)	4.59 (1.72)	0.057
	After intervention	4.99 (1.23)	4.57 (1.06)	0.122
RI	Before intervention	0.95 (0.07)	0.97 (0.05)	0.269
	After intervention	0.95 (0.06)	0.95 (0.07)	0.814
Bladder Volume	Before intervention	25.27 (13.06)	30.56 (15.54)	0.117
	After intervention	50.70 (18.26)	40.48 (19.30)	0.022

*PSV* peak systolic velocity, *TAMX* time average maximum velocity, *EDV* end diastolic velocity, *PI* pulsation index, *RI* resistance index

Data are presented as mean (SD)

### Bladder volume

Before acupuncture, there was no statistically significant difference in bladder volume between the two groups (*P* > 0.05), which was comparable. After acupuncture, the bladder volume of subjects in both groups increased compared with before acupuncture (*P* < 0.05), and the bladder volume of the deep acupuncture group was greater than that of the shallow acupuncture group (*P* < 0.05) (Table 2).

### Anatomical hierarchical structure under BL54

There was no statistically significant difference in the sebum thickness, superficial fascia depth, deep fascia depth, and pudendal nerve depth at the BL54 on the left and right sides of the subjects (*P* > 0.05) (Table 3).

**Table 3:** Analysis of the anatomical hierarchical structure under Zhibian point

Variables	Left (*n* = 74)	Right (*n* = 74)	*P* value
Sebum thickness, mm	3.33 (0.70)	3.39 (0.83)	0.630
Superficial fascial depth, mm	13.11 (2.38)	13.12 (2.50)	0.995
Deep fascial depth, mm	24.78 (5.12)	25.36 (6.28)	0.536
Pudendal nerve depth, mm	58.11 (6.11)	58.11 (6.20)	0.988

Data are presented as mean (SD)

### C-MASS score

After treatment, the C-MASS score of the deep acupuncture group was 42.30 (13.45), and the score of the shallow acupuncture group was 9.03 (6.15), the scale score of the deep acupuncture group was significantly higher than that of the shallow acupuncture group (*P* < 0.01).

### Safety evaluation

Adverse events include needle fainting, broken needles, missing needles, hematomas, infections or abscesses. During this study, no adverse reactions occurred in the two groups of subjects.

## Discussion

The pudendal nerve is one of the main nerves that innervates the pelvic floor structure [10], and injury to the pudendal nerve can lead to pelvic floor dysfunction [11]. Studies have shown that acupuncture stimulation of the pudendal nerve can promote PFM contraction and improve the clinical manifestations of SUI [12]. The pudendal artery is mainly responsible for the blood supply to the PFM, and the vascular status is also closely related to muscle function [13, 14]. Therefore, we speculate that acupuncture stimulation of the pudendal nerve may accelerate the pudendal artery blood flow velocity, thereby improving PFM strength; The pudendal nerve is located below the BL54 and there is no bone obstruction, which facilitates real-time dynamic observation of the entire needle insertion process. Therefore, the BL54 was selected in this study. The PSV and TMAX of the pudendal artery in the deep acupuncture group were significantly greater than before acupuncture (*P* < 0.05), which is consistent with the research results of Mercier et al. who found that PFM training increased pudendal artery blood flow velocity [15], which provides strong evidence that acupuncture can speed up blood circulation to meet the needs of the PFM.

SUI refers to the intravesical pressure is greater than the intraurethral pressure when the abdominal pressure increases during the urinary storage period, and the involuntary outflow of urine [16, 17]. Our study found that after acupuncture, the bladder volume of both groups of subjects increased compared with before acupuncture (*P* < 0.05), and the bladder volume of the deep acupuncture group was greater than that of the shallow acupuncture group (*P* < 0.05). Studies have shown that acupuncture treatment can reduce intravesical pressure, increase bladder capacity [18]. Therefore, we speculate that acupuncture at the BL54 increases bladder volume, reduces intravesical pressure, and thereby reduces urine leakage. The research results of Zhang et al. also prove that acupuncture at the BL54 can increase bladder volume, reduce intravesical pressure[19]. Due to this study detects immediate effects, it is not possible to conduct urodynamic examination and measure changes in bladder pressure, which is also one of the limitations of this study.

After acupuncture, the subjects in the deep acupuncture group felt significantly stronger than the shallow acupuncture group (*P* < 0.05), which is related to the needle tip stimulating different tissue structures of the human body [20]. We have roughly measured the sebum thickness, superficial fascia depth, deep fascia depth, and pudendal nerve depth under the BL54, which can be used as a reference for future similar research and clinical practice.

It is worth mentioning that after acupuncture, the RI of the pudendal artery in the deep acupuncture group showed no significant changes compared with before treatment and the shallow acupuncture group (*P* > 0.05), while the RI of the pudendal artery in the shallow acupuncture group was lower than that before treatment (*P* < 0.05), it is considered that shallow acupuncture of the BL54 have a soothing effect on the pudendal artery and may reduce vascular resistance [21], which requires further exploration.

## Conclusion

Acupuncture at the BL54 can increase the blood flow velocity of the pudendal artery, improve the function of the bladder in controlling urine in women, and different depths of acupuncture will have different therapeutic effects.

## Data Availability

The data is available. If needed, please contact the corresponding author.
